# Health Supplement Consumption Behavior in the Older Adult Population: An Exploratory Study

**DOI:** 10.3389/fpubh.2014.00011

**Published:** 2014-02-10

**Authors:** Mimi Tse, Ka Long Chan, Anthony Wong, Eric Tam, Elaine Fan, Gloria Yip

**Affiliations:** ^1^Center for Gerontological Nursing, School of Nursing, The Hong Kong Polytechnic University, Hong Kong, China; ^2^Interdisciplinary Division of Biomedical Engineering, The Hong Kong Polytechnic University, Hong Kong, China; ^3^Senior Citizen Home Safety Association, Hong Kong, China

**Keywords:** health-related consumption behavior, aging, older adult, health promotion, Hong Kong population

## Abstract

Health supplement consumption behavior is important to maintain health status. The purpose of the study was to explore the spending pattern on health supplement consumption behavior in Hong Kong older adults population. The present study was a cross-sectional survey study; and was collected from via a street-intercept interview. Participants were approached and invited to response to a questionnaire. The location for data collection was evenly distributed in Hong Kong, Kowloon, and New Territories. The questionnaire included demographic data and source of income source, spending habits on health supplement products, and whether they performed regular health check. There were 982 participants interviewed; and 46% was male and 54% was female. The participants are divided into young–old (age 50–69) and old–old group (age 70 or above). The mean age is 67.93 ± 10.386. Most of the participants have regular body check; the major reason is to maintain health. Less than half of the participants spent money on health supplement products; the major reason for such purchase was to maintain health; while for not buying is, they did not think that would have any effect in their health. Also, more young–old participants have regular body check and spend more money on health supplement products; while old–old group participants were less likely to concern their health, and they were less likely to perform regular body check and purchase health supplement products. The present research reveals the pattern of the health supplement consumption behavior of young–old and old–old. Young–old group and old–old group have difference pattern according to their difference age-related health condition and the amount of spare money. Different educational program concern health consciousness and promotion strategy of regular body check and health supplement products need be tailor-made for older adults, and for young–old and old–old groups.

## Introduction

According to the World Health Organization (WHO), the aging population is increasing faster than any other (1). The population aged 65 and over is expected to increase by 180% between 2010 and 2050 as compared to an anticipated 32% increase in the population aged between 15 and 64 (2). Hong Kong is no exception to this global trend. According to the Census and Statistics Department, HKSAR, the proportion of the population aged 65 and over will increase from 13% in 2011 to 30% in 2041. By contrast, the population aged 15–64 will decrease from 75 to 61% in the same period (3). There is a negative relationship between health and age (4), thus, older adults have worse health status than younger adults. A report in 2013 reveals that the population aged 65 and over in Hong Kong has the poorest health status of all age groups, based on a self-perceived general health condition questionnaire (4). They also have the highest proportion of individuals suffering from chronic health conditions, at 73% (4). The four most common chronic diseases are hypertension (62%), diabetes mellitus (27%), high cholesterol (18%), and heart disease (13%) (4). Growing older can therefore be said to contribute to declining health.

The WHO Ottawa Charter for Health Promotion introduced the concept of health promotion, defined as “the process of enabling people to increase control over, and to improve, their health.” Health is seen as a resource for everyday life beyond the responsibility of the health section as a way to achieve well-being. Good health is a major resource for social, economic, and personal development and an important dimension of quality of life. Professional and social groups and health personnel have a major responsibility to mediate between differing interests in society for the pursuit of health. Health promotion focuses on achieving equity in health (5). Specific health promotion behaviors and practices would include living a healthy lifestyle, receiving regular physical checkups, and using health supplement products.

Health supplement consumer behavior refers to buying health supplement products and receiving physical checkups. Several studies have examined health supplement consumer behavior, but they did not focus on older adults. Research shows that those with higher health consciousness are more willing to use functional foods (6), that the high nutritional value of food is the most important factor in determining their health food purchases (7), that perceived behavioral control and attitude toward healthy eating are the most important predictors of healthy eating behavior in adolescents (8), and that advertisements from medical professionals are useful to consumers in buying health supplement products (9). However, there is a lack of research on older adults’ health supplement consumer behavior.

Health supplement consumer behavior is important to older adults; they can maintain and enhance their health status through purchasing health food, and get regular physical checkups to monitor their health condition. However, aging has an effect on cognition, thus older and younger adults have different cognitive ability (10). Older adults have impaired processing speed, working memory, and long-term memory, thus they have different ways of processing the information they receive. Only self-perceived important information and that requiring fewer cognitive resources can get processed in older adults. Thus, older adults and younger adults behave differently when it comes to decision making, particularly as regards consumer behavior.

Because older adults have a different decision-making processes, there is a knowledge gap in terms of health supplement consumer behavior as it applies to older adults. Thus, there is a need to examine the underlying process of older adults’ decision making in this area. Based on evidence from the empirical study, messages and advertisements about health supplement products can be framed for older adults, and strategies on health supplement product marketing can be tailored to older adults. Therefore, the aim of this research was to explore the Hong Kong older adult population’s spending patterns on health supplement products and their usage of regular physical checkups.

## Materials and Methods

### Data collection

This was a cross-sectional survey study, with older adults’ consumer behavior data collected from a street-intercept questionnaire. The target was those aged 50 or over; participants were divided into a young–old and an old–old group according to their age, either 50–69 or 70 and over, respectively. The Hong Kong government offered an old age allowance for Hong Kong residents aged 70 and above, therefore we set the cut-off age as 70. The interview locations were in private housing complexes in Hong Kong, Kowloon, and the New Territories. The sample size was based on data from the Census and Statistics Department in 2011 (11); the total population of Hong Kong was 7,070,338, of whom 35.4% (*n* = 2,501,105) were aged 50 or above. By setting α = 0.01 and with a 5% of margin of error, the estimated sample size was 609. Using proportional sampling, the sample sizes for Hong Kong, Kowloon, and the New Territories were 118, 193, and 298, respectively. The target population was approached individually, and they were invited to join our study by answering the questionnaire administered by the research team. The estimated time needed to complete the questionnaire was around 10 min.

### Measures

The questionnaire comprised three parts, of which the first elicited the subject’s demographic data, including age, gender, marriage status, education level, income source, and living status. The second covered expenditure, and consisted of two questions: “After deducting basic expenses, what is your monthly disposable income?” and “Do you have enough money to pay for your daily necessities?” The third part covered health supplement consumer behavior and its definition included having regular physical checkups and purchasing health supplement products. Examples of health supplement products included health supplement food (cod liver oil, ganoderma spores, calcium tablets), medical equipment (blood pressure and blood glucose measurement devices), and drugs. It contained three questions, “Do you have regular physical checkups?” “Do you purchase health supplement products?” and “Do you introduce health supplement products to others?”. Respondents were asked to give their reasons after answering each question. Questions were closed-ended with multiple choice answers from which respondents were asked to choose the most suitable answers.

### Data analysis

The statistical software SPSS (Version 20) was used to process and analyze the quantitative data. Intention-to-treat analysis was conducted for any missing data. Descriptive statistics were generated to illustrate the characteristics of the young–old and old–old group participants. The Chi-squared test was used to compare the group differences. A *p*-value of <0.05 was considered statistically significant for all statistical testing.

## Results

### Demographic data

As shown in Table 1, nine hundred eighty-two older people, 46% male and 54% female, participated in this interview. The participants were divided into a young–old and an old–old group. The mean age was 67.93 ± 10.386. The sampling locations were in private housing estates in Hong Kong, Kowloon, and the New Territories (19, 32, and 49%, respectively). Most of the participants were married (81% for the young–old and 68% for the old–old group). In terms of education, 72% of the young–old participants had reached secondary level, while only 42% of the old–old participants had reached secondary level.

**Table 1:** **Demographic data**.

	Total (*N* = 982)	50–69 (*N* = 493)	70 and over (*N* = 488)	*p*-Value
	*N* (%)	*N* (%)	*N* (%)	
Age	67.93 (10.386)			
Gender
Male	445 (45.6)	228 (46.6)	217 (44.6)	0.517
Female	531 (54.4)	261 (53.4)	270 (55.4)	
Marriage status
Married	721 (74.8)	390 (81.1)	331 (68.5)	0.000**
Divorce	119 (12.3)	47 (9.8)	72 (14.9)	
Single	40 (4.1)	29 (6.0)	11 (2.3)	
Widowed	84 (8.7)	15 (3.1)	69 (14.3)	
Interview location
Hong Kong	186 (19.0)	94 (19.1)	92 (18.9)	0.446
Kowloon	311 (31.7)	148 (30.0)	163 (33.4)	
New Territories	484 (49.3)	251 (50.9)	233 (47.7)	
Education level
No formal education	141 (14.4)	9 (1.8)	132 (27.3)	0.000**
Primary level	275 (28.2)	127 (25.8)	148 (30.6)	
Secondary level	404 (41.4)	248 (50.4)	156 (32.2)	
Tertiary level or above	156 (16.0)	108 (22.0)	48 (9.9)	
Income source^a^
Supported by children	524 (53.4)	194 (39.4)	330 (67.6)	0.000**
OAA	338 (34.5)	20 (4.1)	318 (65.2)	
Working	235 (24.0)	223 (45.2)	12 (2.5)	
Savings	236 (24.1)	116 (23.5)	120 (24.6)	
Pension	122 (12.4)	65 (13.2)	57 (11.7)	
Investment	88 (9.0)	59 (12.0)	29 (5.9)	
Supported by partner	48 (4.9)	45 (9.1)	3 (0.6)	
CSSA	26 (2.7)	2 (0.4)	24 (4.9)	
Living status
Living with family member	258 (26.4)	89 (18.1)	169 (34.6)	0.000**
Private rental housing	62 (6.3)	38 (7.7)	24 (4.9)	
Own housing	515 (52.6)	299 (60.9)	216 (44.3)	
Public rental housing	138 (14.1)	63 (12.8)	75 (15.4)	
Other	6 (0.6)	2 (0.4)	4 (0.8)	

*Chi-square was used*.

**p < 0.01 was considered to be statistically significant.

*^a^ Multiple responses*.

In terms of income sources, most of the young–old participants relied on the salary from their job, support from their children, and their savings (45, 39, and 23%, respectively), while the old–old participants relied on support from their children, OAA, and their personal savings (67, 65, and 24%, respectively).

Regarding living status, most of the participants lived in privately owned housing (60% for the young–old group and 44% for the old–old group), while 18% lived with family members, 12% lived in public rental housing, and 7% lived in private rental housing, respectively, among the young–old group, which was similar to the old–old group (34, 15, and 4%, respectively). Overall, there were statistically significant differences between the two groups in terms of marriage status, education level, income source, and living status.

### Expenditure

In terms of disposable income, 21% of participants in the young–old group had <$1500 per month, 27% had $1500–$2999, 19% had $3000–$4999, and 30% had more than $4500 after deducting their necessary living expenses. In the old–old group, 39% of participants had <$1500 of disposable income, 30% had $1500–$2999, 11% had $3000–$4999, and only 16% had more than $4500 per month. The percentage of disposable income was statistically significantly different between the two age groups, *X*^2^(4, *N* = 982) = 60.31, *p* < 0.01 (see Table 2).

**Table 2:** **Expenditure**.

	Total (*N* = 982)	50–69 (*N* = 493)	70 and over (*N* = 488)	*p*-Value
	*N* (%)	*N* (%)	*N* (%)	
After deducting basic living expenses, how much money is left for you to spend each month?				
$1500 or less	294 (30.0)	104 (21.1)	190 (39.1)	0.000**
$1500–$2999	284 (29.0)	136 (27.6)	148 (30.5)	
$3000–$4499	154 (15.7)	96 (19.5)	58 (11.9)	
$4500 or more	231 (23.6)	152 (30.8)	79 (16.3)	
Unclear	16 (1.6)	5 (1.0)	11 (2.3)	
Do you have enough money to supply the necessities of life?				
Not enough, and living is difficult	78 (8.0)	40 (8.2)	38 (7.9)	0.010*
Barely enough	395 (40.6)	179 (36.6)	216 (44.6)	
Enough, have money left over	380 (39.1)	195 (39.9)	185 (38.2)	
More than enough	120 (12.3)	75 (15.3)	45 (9.3)	

*Chi-square was used*.

*p < 0.05 and **p < 0.01 were considered to be statistically significant.

Regarding the adequacy of money for their daily necessities, 8% of the young–old group did not have enough money, 36% had barely enough, 39% had enough, and 15% had more than enough money. In the old–old group, 7% of participants did not have enough money, 44% had barely enough, 38% had enough, and 9% had more than enough money. The young–old and old–old had a statistically significant difference in the adequacy of money, *X*^2^(3, *N* = 982) = 11.26, *p* = 0.01 (see Table 2).

### Health supplement consumption behavior

In terms of physical checkups, most of the participants had them regularly (73% for the young–old and 80% for the old–old group). The frequency of their checkups were statistically significantly different between the two age groups, *X*^2^(1, *N* = 982) = 8.26, *p* < 0.01. The major reason for having regular checkups was to maintain health (73% for the young–old and 64% for the old–old group), and because it was requested by others (10% for the young–old and 20% for the old–old), while the major reasons for not having regular physical checkups were that they were not needed (74% for the young–old and 61% for the old–old), and cost too much (14% for the young–old and 23% for the old–old). Overall, young–old and old–old were statistically significantly different in their reasons both for having regular checkups, *X*^2^(5, *N* = 982) = 23.27, *p* < 0.01 and for not having regular checkups, *X*^2^(5, *N* = 982) = 7.54, *p* = 0.18 (see Table 3).

**Table 3:** **Health supplement consumption behavior**.

	Total (*N* = 982)	50–69 (*N* = 493)	70 and over (*N* = 488)	*p*-Value
	*N* (%)	*N* (%)	*N* (%)	
Do you have regular physical exams?^a^				
Yes, reason(s)	756 (77.1)	361 (73.2)	395 (80.9)	0.004**
Health	517 (69.2)	266 (73.9)	251 (64.9)	
Requested by others	114 (15.3)	36 (10.0)	78 (20.2)	
Habit	87 (11.6)	42 (11.7)	45 (11.6)	
Low cost	59 (7.9)	26 (7.2)	33 (8.5)	
Convenience	6 (0.8)	4 (1.1)	2 (0.5)	
No, reason(s):	225 (22.9)	132 (26.8)	93 (19.1)	
No need	150 (69.1)	95 (74.2)	55 (61.8)	
High cost	39 (18.0)	18 (14.1)	21 (23.6)	
No time	29 (13.4)	16 (12.5)	13 (14.6)	
Fear	9 (4.1)	6 (4.7)	3 (3.4)	
No method	3 (1.4)	2 (1.6)	1 (1.1)	
Do you purchase health supplement products?^a^				
Yes	372 (37.9)	218 (44.2)	154 (31.6)	0.000**
Health supplement food	232 (62.9)	150 (69.4)	82 (53.6)	
Medical equipment	159 (43.1)	86 (39.8)	73 (47.7)	
Drugs	10 (2.7)	1 (0.5)	9 (5.9)	
No	609 (62.1)	275 (55.8)	334 (68.4)	
Do you introduce health supplement products to others?				
Yes	151 (41.5)	111 (52.4)	40 (26.3)	0.000**
No	213 (58.5)	101 (47.6)	112 (73.7)	

*Chi-square was used*.

**p < 0.01 was considered to be statistically significant.

*^a^ Multiple responses*.

Regarding the purchase of health supplement products, fewer than half of the participants spent money on these (44% for the young–old and 31% for the old–old group). The percentage of purchasing health supplement products among participants was statistically significantly different between the two age groups, *X*^2^(1, *N* = 982) = 16.70, *p* < 0.01. Expenditure on health supplement products made up 69% of health expenditure for the young–old and 53% for the old–old group, and that on medical equipment represented 39% of health spending for the young–old and 47% for the old–old group. These frequencies were statistically significantly different across the two age groups, *X*^2^(3, *N* = 982) = 21.89, *p* < 0.01 (see Table 3).

As regards introducing health supplement products to others, 52% of young–old participants had done so, compared with only 23% of old–old participants. The relationship between introducing health supplement products to others and age group was statistically significant, *X*^2^(1, *N* = 982) = 24.73, *p* < 0.01. The old–old were less likely to introduce health supplement products to others (see Table 3).

### Reasons for purchasing health supplement products

In terms of their reasons for buying health supplement products, most of the participants did so for health maintenance (68% for the young–old and 63% for the old–old group), while the less major reasons were introduction by family members or friends (20% for the young–old and 24% for the old–old group) and introduction by medical professionals (16% for the young–old and 26% for the old–old). These frequencies were statistically significantly different between the two age groups, *X*^2^(7, *N* = 982) = 14.90, *p* = 0.04 (see Table 4).

**Table 4:** **Reasons for buying/not purchase health supplement products**.

	Total (*N* = 982)	50–69 (*N* = 493)	70 and over (*N* = 488)	*p*-Value
	*N* (%)	*N* (%)	*N* (%)	
Why do you purchase health supplement products?^a^				
Health	247 (66.4)	149 (68.3)	98 (63.6)	0.037*
Recommended by family members or friends	81 (21.8)	44 (20.2)	37 (24.0)	
Recommended by medical professionals	76 (20.4)	36 (16.5)	40 (26.0)	
Family members or friends have bought	39 (10.5)	23 (10.6)	16 (10.4)	
Advertisement	38 (10.2)	28 (12.8)	10 (6.5)	
Low cost	4 (1.1)	4 (1.8)	0	
Habit	2 (0.5)	2 (0.9)	0	
Why do you not purchase health supplement products?^a^				
Not effective	279 (46.3)	140 (51.7)	139 (41.9)	0.107
Too costly	143 (23.7)	58 (21.4)	85 (25.6)	
No faith in the products	96 (15.9)	36 (13.3)	60 (18.1)	
Perceived good health already	56 (9.3)	29 (10.7)	27 (8.1)	
No need	55 (8.8)	26 (9.6)	27 (8.1)	
Do not want to rely on health food	30 (5.0)	12 (4.4)	18 (5.4)	
Do not want to eat health food	18 (3.0)	6 (2.2)	12 (3.6)	
Offered by hospital or clinic	9 (1.5)	3 (1.1)	6 (1.8)	
Not recommended by medical professionals	8 (1.3)	2 (0.7)	6 (1.8)	

*Chi-square was used*.

*p < 0.05 was considered to be statistically significant.

*^a^ Multiple responses*.

Regarding their reasons for not buying health supplement products, in the majority of cases, it was because they believed that the products had no effect (51% for the young–old and 41% for the old–old), while the less major reasons were high cost (21% for the young–old and 25% for the old–old), and uncertainty regarding the effect of the products (13% for the young–old and 18% for the old–old). These frequencies were not statistically significantly different between the two age groups, *X*^2^(7, *N* = 982) = 14.46, *p* = 0.11 (see Table 4).

## Discussion

The present study investigated the health supplement consumer behavior of people aged 50 and above, mainly in terms of receiving regular physical checkups and purchasing health-related products. The findings show that the amount of disposable income may be not related to the adequacy of income. Table 2 shows the difference between the two groups in the amount of available spending money, with the young–old group nearly equally distributed across the four spending money brackets, while the majority of the old–old group had <$3000 available per month after meeting living expenses. This suggests that the old–old group has less spending money available than the young–old group. However, there were similar patterns in the sufficiency of money among the two groups: both were concentrated in the “barely enough” and “enough” categories. Although the amount of available spending money differed, this did not influence their perception of its adequacy. One possible reason is that old–old participants reduce their expenses in order to manage the necessities of daily life. They do this by lowering their quality of life, avoiding unnecessary expenses, and reducing the number of entertainment activities. Another reason is that they rely on other subsidies to maintain their daily life; this includes support from their children, necessities, and subsidies from the government and other NGOs.

Health maintenance was the major reason given for undergoing regular physical exams and buying health supplement products (see Table 3). Although a higher percentage of the old–old group received regular checkups, fewer of them cited health maintenance and more of them cited others’ request as their reasons. Older adults are in worse health condition (4), which may lead to a higher chance of their seeking regular physical exams. This would suggest that health is not the main concern in the old–old group when compared with the young–old group. In addition, autonomy declines with increasing age (12), thus older adults have a greater chance of having their decisions made by others. The major reasons given by participants for not having regular physical checkups or buying health supplement products were lack of need and high cost (see Tables 3 and 4). Also, a higher percentage of the old–old group cited high cost as their reason for not having regular physical checks and not buying health supplement products. This would explain the phenomenon, whereby, although the old–old group has less spending money available, they still consider themselves to have enough for the necessities of daily life. Two of the expenses they tend to eliminate are regular health checks and health supplement products, suggesting that the old–old group does not perceive these to be necessities of daily life.

As shown in Table 3, there was a difference between the two groups in terms of introducing health supplement products to others. A higher percentage of the young–old group would do so compared to the old–old group. One reason is that the old–old group is more focused on purchasing medical equipment and drugs than the young–old group: because medical equipment and drugs are specific to different patients with different needs and concerns, they are more difficult to share with others than, for example, health food, which is not tailor-made and more likely to be of interest to others. Another reason is that older adults have poor health condition and mobility (4, 13), causing them to engage in fewer social activities (14, 15) and limiting their social activities to meeting a few people in the park, the older adult community center, and the immediate neighborhood. This may limit their ability to introduce health products and equipment to others. Because of this and the nature of the products they use, the old–old group is less likely to introduce health products to others.

Although fewer among the old–old group tended to introduce products to others, they were more likely than the young–old group to try products that others introduced to them. One possible explanation is that the old–old group has impaired decision-making ability (10), and information regarding health-related products is too complex, thus they tend to rely on the recommendations of others. This is in contrast to some research (16) suggesting that conformity decreases with increasing age. However, the present research focuses on a type of decision-making that requires high cognitive ability to analyze; if the process of analysis fails, they rely on other people’s recommendations.

The major reasons for not undergoing regular physical exams and not buying health supplement products were the perceptions that they did not need them and that the products were not effective, or that the person had no faith in them. This suggests that information regarding a product is not always distributed to participants, and also that there was no heuristic to facilitate the decision-making process. Until people have enough information about a product to ensure that it is safe, useful, and effective, or until they have a self-perceived reliable heuristic, they prefer to do nothing (17).

The present research findings suggest that the stereotype of health supplement consumer behavior needs to be changed when marketing products and drugs to the old–old group. The concept of active aging, as introduced by the WHO, considers aging as an active process in spite of its inevitability (18). Under this framework, health is still a major concern to older adults, more so than to younger adults as older adults are in comparatively poorer health condition (4). To maintain health, regular physical checkups and health supplement products are necessities of daily life. However, because of the nature of aging, information needs to be framed for two separate groups. For the young–old, information can be presented in a more detailed way through appealing to reason, while for the old–old, information can be presented in more simple terms, emphasizing the need for the product; the old–old needs to be persuaded by appealing to their emotions (17). The costs of regular checkups and health supplement products need to be reduced for both groups according to the results of the present research in regard to available spending money.

## Summary

This study reveals the patterns of health supplement consumer behavior among the young–old and the old–old. The young–old and old–old have different patterns according to their different age-related health conditions and the amount of spending money available to them. Different educational programs concerning health consciousness and the promotion strategy of regular health checks and health-related products need to be tailored to young–old and old–old groups.

## Conflict of Interest Statement

The authors declare that the research was conducted in the absence of any commercial or financial relationships that could be construed as a potential conflict of interest.
